# Supplementary figures and images for: Cryo-EM structures reveal two distinct conformational states in a picornavirus cell entry intermediate
Source: PLoS Pathog. 2020 Sep 30;16(9):e1008920. doi: 10.1371/journal.ppat.1008920 (PMC7549760; doi:10.1371/journal.ppat.1008920)

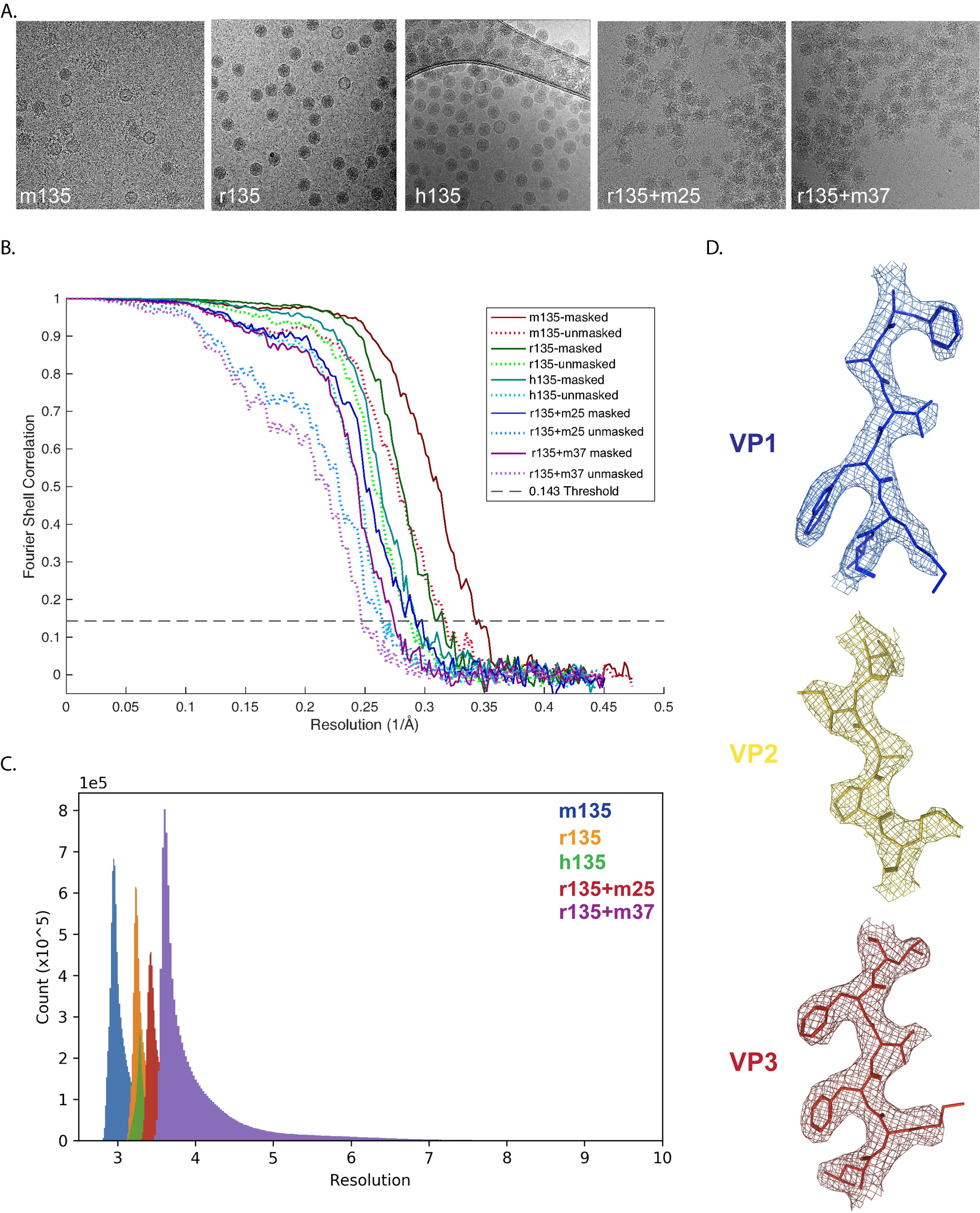

Supplement: S1 Fig — (A) Representative, raw micrographs of the datasets used in the analysis, (B) Masked (continuous line) and unmasked (dotted line) Fourier shell correlation plots of m135 (red), r135 (green), h135 (cyan), r135+m25 (blue) and r135+m37 (purple). At a 0.143 cut-off level, the resolution estimates range from 2.8 to 3.6Å. (C) Local resolution estimates in all the maps were calculated using Relion and the distribution of resolution values within in the masked region is plotted. (D) In the best resolved dataset (m135), individual amino acid side chains are easily discriminated. (TIF) [file ppat.1008920.s001.tif]

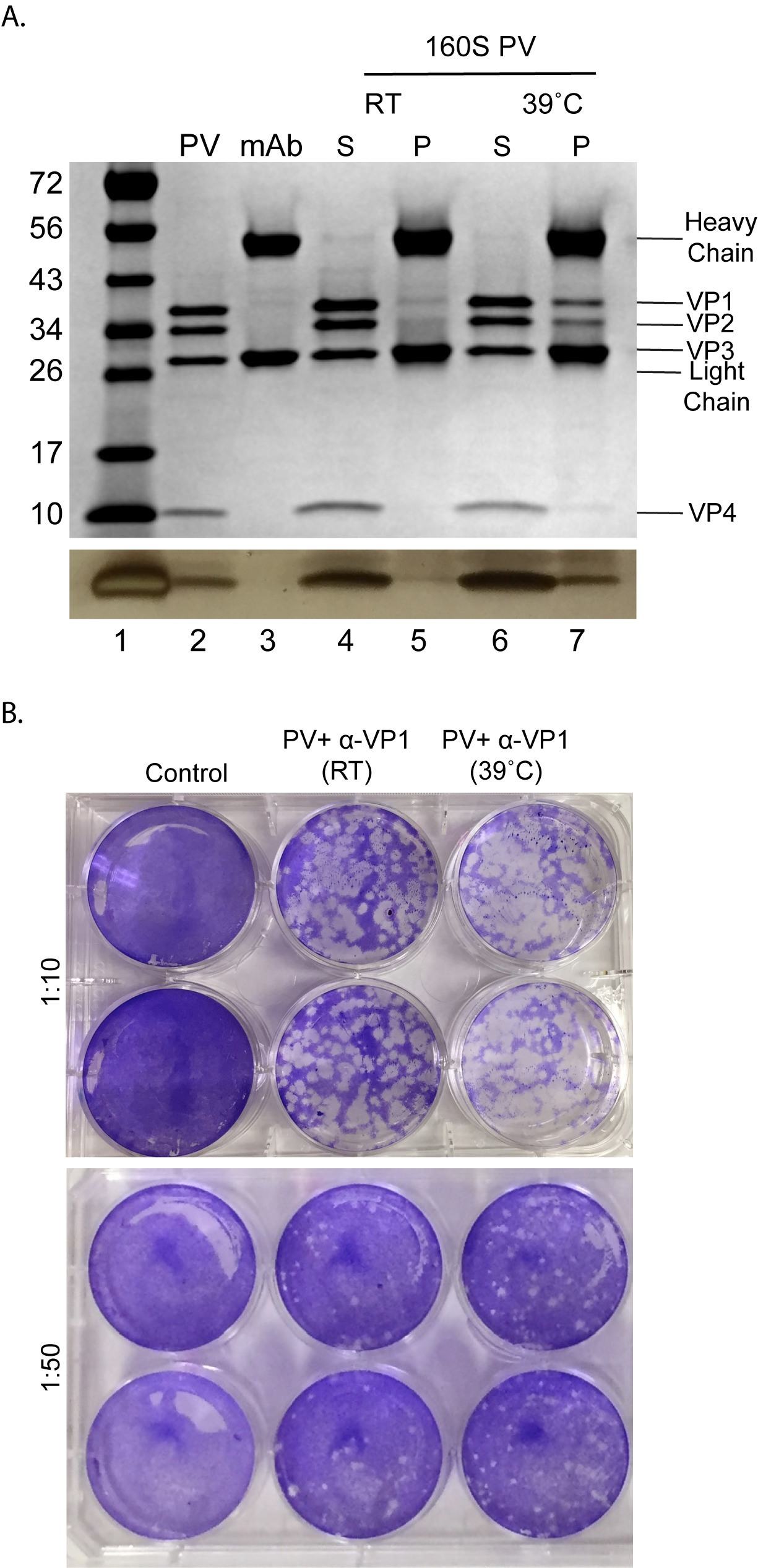

Supplement: S2 Fig — (A) The anti-VP1 antibody specifically recognizes and binds the N-terminus of VP1 only after the N-terminus has been externalized at 39°C. Poliovirus particles were complexed with the antibody at room temperature or at 39°C for 1.5 h and immunoprecipitated with magnetic Protein A coated beads. After thorough washing of the beads to remove unbound material, the samples were examined on SDS-PAGE gels. Lane 1, Ladder; lane 2, PV particles only; lane 3, antibody; lane 4, soluble fraction of antibody plus 160S PV after binding at RT; lane 5, pelleted fraction of antibody plus 160S PV after binding at RT; lane 6, soluble fraction of antibody plus 160S PV after binding at 39°C; lane 7, pelleted fraction of antibody plus 160S PV after binding at 39°C. Bottom panel, silver staining of the gel to enhance the relatively weak VP4 signal (B) Expanded poliovirus particles are infectious. Native (160S) poliovirus particles previously incubated with anti-VP1 antibody at room temperature or at 39°C for 1.5 h were immunoprecipitated with magnetic Protein A coated beads. After thorough washing of the beads to remove unbound material, the beads were freeze-thawed to release poliovirus particles. Released poliovirus was serially diluted and plated on naïve Vero cells. After 36 h of incubation at 37°C, plaques were visualized by staining with crystal violet. (TIF) [file ppat.1008920.s002.tif]

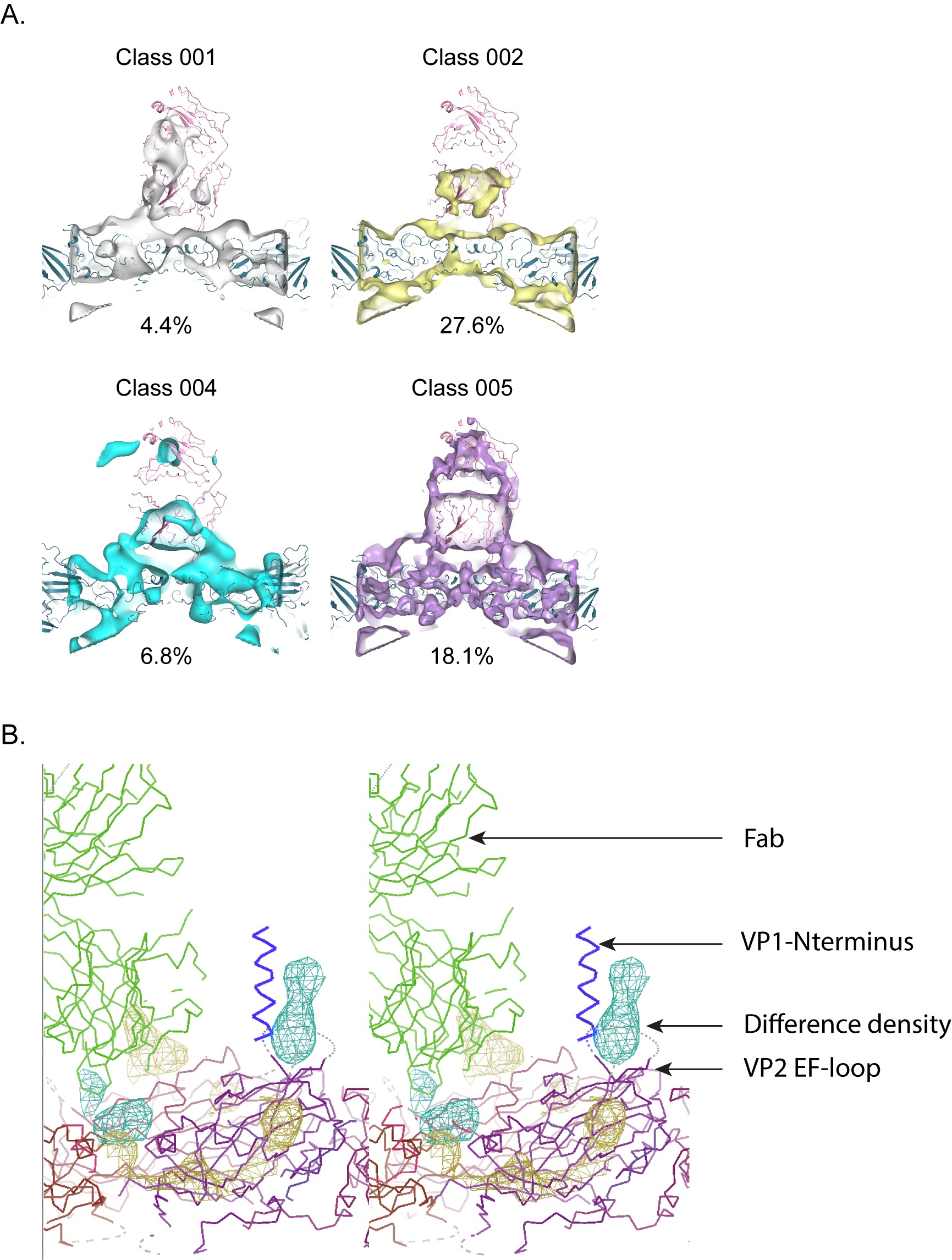

Supplement: S3 Fig — (A) Asymmetric focused classes calculated for the r135+m25 dataset with percentage population per class. Class3 is depicted in Fig 4B and 4C. (B) Difference density, shown here in stereo, was calculated from previously published low-resolution reconstructions of poliovirus 135S particles, either untreated or treated with V8 protease, which cleaves at residue 31 of VP1. As a guide, we have superimposed an alpha carbon model of r135+m25. The blue alpha helix (putatively residues 1–21 of VP1) was fitted to a focused class of r135+m25 (as shown in Fig 4D and 4E). Observe that the putative VP1 helix model, bound to the top of VP2, is similar in position and orientation to the previously reported difference map feature. (TIF) [file ppat.1008920.s003.tif]

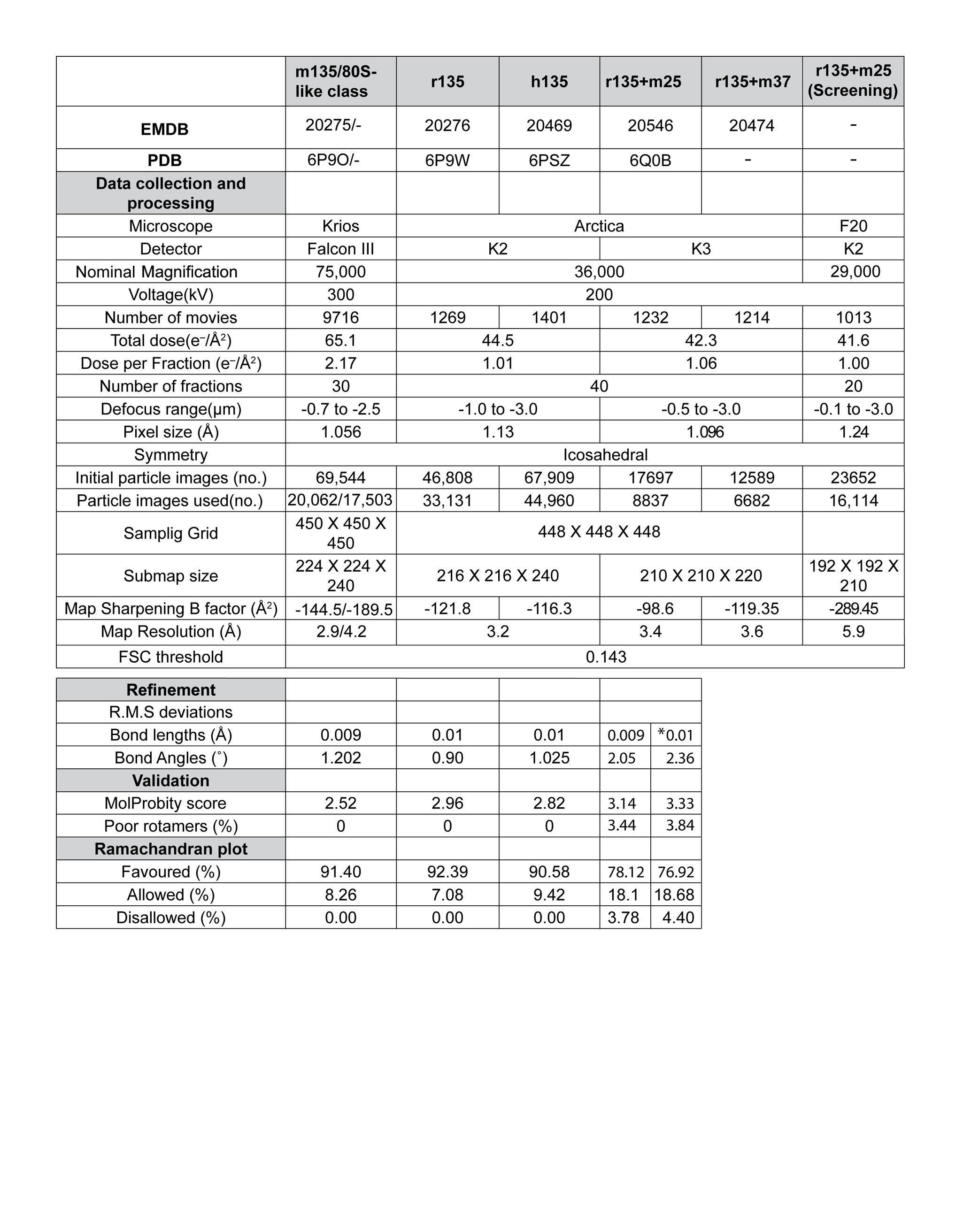

Supplement: S1 Table — *The r135+m25 model, which includes placeholders for the Fab and VP4 densities, was calculated with phenix_refine, using its automatic choice of weighting schemes. Other final atomic models were refined with Refmac5, using stronger stereochemical restraints. (TIF) [file ppat.1008920.s004.tif]

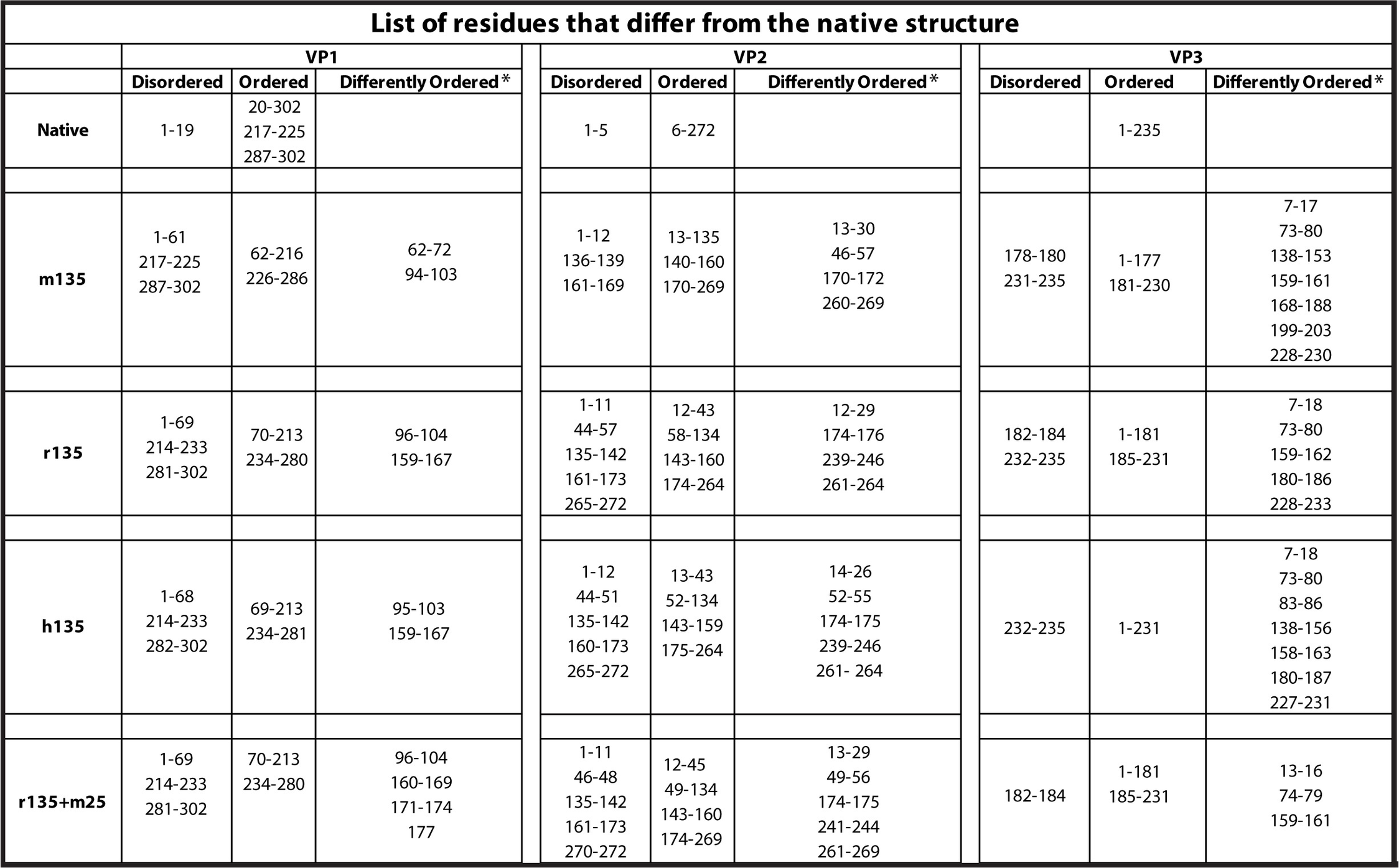

Supplement: S2 Table — *Differently ordered residues are those residues that significantly differ in position from the native model (1HXS) following a least squares superposition of individual capsid proteins. (TIF) [file ppat.1008920.s005.tif]
