# Supplementary material for: Cryo-EM structures reveal two distinct conformational states in a picornavirus cell entry intermediate
Source: PLoS Pathog. 2020 Sep 30;16(9):e1008920. doi: 10.1371/journal.ppat.1008920 (PMC7549760; doi:10.1371/journal.ppat.1008920)
Supplement: Attachment — Submitted filename: ShahPNMS_et_al_Response2Reviewers.pdf [file ppat.1008920.s006.pdf]

We thank the editor and reviewers for their careful attention to our manuscript. In responding to the comments and questions, we have addressed each point raised by the two reviewers as outlined below.

Both reviewers raised questions about our tentative assignment of a band of density inside the capsid shell, which had been assigned to the viral RNA in other structures, as partially occupied VP4. As outlined in our response we believe that the biochemical evidence that significant levels of VP4 remains associated with poliovirus 135S particles is rock solid, that, given that VP4 plays a significant role in infection, the presence of VP4 in the 135S particles is virtually required to explain the fact that these particles are infectious, and that the position and shape of the density are more consistent with it being assigned to VP4. As outlined below we have made several changes in the manuscript to clarify these points but stand by our tentative assignment. We hope that the editor will agree that the case for this identification, which we believe we have sufficiently couched as speculative, is sufficiently strong to warrant publication as an alternative explanation that should be considered in future structural studies.

The editor pointed out that at several places in the manuscript we had referred to “data not shown” and pointed out that this is not allowed by journal policy in order to prevent significant findings from being presented without showing supporting data.

Author Response:

In every case where the phrase “data not shown” was used in the manuscript, the data from the experiment could be and were described in a simple declarative phrase, and the use of the phrase was therefore actually superfluous and could be eliminated. Thus, we have deleted the phrase “data not shown” from the following places in the manuscript:

Lines 134-136: “Initial screening of conditions to trap the N-terminus outside the virus outside the virus using the mAb at 37°C yielded very few decorated particles (data not shown).”

Lines 136-140: “Therefore, we performed addition cryo-EM screenings of the virus-mAb complexes for durations of up to 120 minutes at temperatures of 38°and 39°C (data not shown). An optimal number of complexes was observed after a 90-minute incubation of the virus with the mAb at 39°C.”

Lines 490-491: “produced a fenestrated reconstruction that lacked significant density for the VP1 N-terminus (data not shown).”

Lines 598-601: "Asymmetric focus maps were calculated for cylindrical volumes near the 2-fold axes to see if any well-ordered Fab binding sites had been smeared out by symmetry-based averaging, but no such well-ordered sites were seen (data not shown)."

We feel that the reviewers' comments and the responses we have made to address them have improved the manuscript and hope that it is now ready to move forward with publication in PLoS Pathogens.

Reviewer #1:

1. Many other studies have reported the "early" state of uncoating intermediates through either receptor inducing or acid treatment, e.g. the novel expanded (E1) EVD68 particles (Liu et al, 2018, PNAS). The author should mention the difference between the early 135S-like PV in this study to E1 particle (in case of EVD68) or some other receptor-induced intermediates? The paper should cite these papers and discuss with them properly.

Author Response:

The reviewer brings up two points in this comment: 1) That a paper describing the structure of the 135S particles of enterovirus D68 was not cited, 2) that we had not adequately addressed the similarity of the poliovirus 135S structures we report here with structure of the 135S particles of EVD68 and other enteroviruses.

We thank the reviewer for bringing the omission of a citation of the EVD68 structure. The omission was inadvertent but significant because the paper documents an observation of a minority population of the D68 135S particles that are expanded but have the VP1 N-terminus totally inside the particle similar to our observations in the h135 and r135 poliovirus particles We have added the reference to the EVD68 135S structures at several places as noted in the next paragraph and in particular have pointed particles with internal VP1 N-termini in the sections of the results **(lines 307-314)** and discussion **(lines 480-481**) sections of the manuscript. The insertion at **lines 307-314** (see below) specifically discusses how the D68 E1 particle is consistent with and extends the idea that the formation of the 135S particle is a multi-step process.

Although we had cited the other structures at various places in the manuscript to point out differences between structures, the reviewer is correct in pointing out that this did not quite accomplish our goal of putting the structures in context, and in particular to point out how remarkably similar the structures actually are. We have added additional citations to either all of the structures, or subgroups of the structures to point out areas where they are similar at several places in the manuscript: lines 87-89, line 189, lines 213-216, 232-235, and line 539.

Lines 87-89: “These openings permit the externalization of the membrane-interactive N-terminal extension of capsid protein VP1 [11,14] in poliovirus and in the 135S particles of other enteroviruses [19-26] and presumably VP4, which is myristoylated [27]”

Line 187: “Similar to 135S-like particles for other enteroviruses [19-26],”

Lines 213-214: “and is strikingly similar to the shifts seen in the structures of 135S particles of other enteroviruses [19-26]”

Lines 233-234: “previously published for poliovirus and other enteroviruses [14,18-20,23-26,41] (see ordered residue lists, Table S2).”

Lines 302-311: “Interestingly, an expanded particle in which the VP1 N-terminus remained inside the capsid has also been observed as a minority subpopulation (~5% of the particles) in preparations of 135S particles of enterovirus D68 [19]. However, in this subpopulation of the D68 135S particles the entire VP1 N-terminal extension is well ordered suggesting that the “early” state of the 135S-like particles may itself be comprised of two steps. Thus, in a first step the particle undergoes expansion with the VP1 N-terminus remaining in a well-ordered native-like arrangement on the inner surface of the expanded capsid; this is followed by a second step in which the VP1 N-terminal extension dissociates from the inner surface, prior to being externalized.”

Lines 476-478: “Expanded particles with internal (but ordered) VP1 N-termini have also been observed as a minority population in preparations of 135S particles of enterovirus D68 [19].”

Line 536: “and in the structures of the 135S particles of other enteroviruses [19,20,22-26].”

2a. On the other hand, one of important feature of this early state of intermediate is that VP4 remains inside the particle and accounts for a feature that had been previously ascribed to part of the viral RNA. However, this claim seems take a risk

in terms of cryo-EM structure at median resolution. There is no direct biochemical evidence supporting the presence of VP4 in the expanded particles.

As we explain in the manuscript, the continued association of VP4 in 135S particles was established by Curry et al. Additionally, the fact that 135S is infectious (Curry et al.), and our demonstration of mAb-triggered expansion being reversible (see Supplement) both imply that VP4 is present in the sample, in association with the virus.

We have revised the text to make the findings of the Curry et al. paper more obvious (**lines 444-455**)

Lines 444-455: "The proposed identification is supported by the findings of Curry et al. [17], which clearly showed low levels of VP4 (up to 25% of the levels observed in virions) in highly purified preparations of poliovirus 135S particles. That study disproved the widely held claim that VP4 (which stains and radiolabels poorly and is difficult to detect in SDS gels optimized for the larger capsid proteins VP1-3) was totally lacking in 135S particles. Moreover, because VP4 plays a key role in viral infection [47], at least some VP4 must remain present in 135S particles to explain the observation that these particles remain infectious [17,18]. Together with the biochemical evidence, the position and shape of the density feature suggests that the thin layer immediately adjacent to the capsid that has previously been attributed to RNA is more likely to be residual VP4 pentamers that are not externalized until the RNA genome is released."

2b. Although the density (10Å thick) is pentagonal and looks thinner rather than duplex RNA, a possibility is with artefact of the reconstruction calculation with icosahedral symmetry imposed.

We agree that the identification of VP4 is somewhat speculative (and point this out ourselves in the manuscript **line 422-424**). We argue, nevertheless, that the existing picornavirus literature repeatedly (and possibly mistakenly) identifies the same density feature as RNA, based on even weaker evidence than we are presenting. We believe that the literature would benefit from a presentation of this alternative view.

Beyond the coincidence that the size and shape of the low-resolution feature seem appropriate for VP4 pentamers, and inappropriate for RNA, we call attention to the remarkable fact that the feature occupies the same radial position as VP4 pentamers do in native virus (suggesting continued association with the "RNA ball"), while the inner N-terminal network changes, and the larger capsid proteins expand outward by a highly reproducible amount (as established unambiguously with high resolution density). This mechanistic description is novel, attractive, and could have important implications for our future understanding of how picornavirus uncoating works.

As for the imposition of symmetry, we did, indeed, attempt non-symmetric focused refinements of the putative RNA density, and observed that explicit symmetry restraints were not required to get the pentameric density feature to maintain its general shape. However, they never achieved a resolution sufficient to show individual polypeptide chains or improved interpretability so they were not reported in the manuscript.

2c. Additionally, the description of visualising the exposed N-terminal helix of VP1 should be careful in terms of a low local resolution.

The identification of the helix density was not based on the density appearance alone but was confirmed by other lines of evidence. We believe that the evidence supporting the N-term helix identification, taken together, is fairly strong. The cylindrical shape of the density, indicating a helix, is distinctive, and the shape is well-fit by an idealized helix with the sequence of the putative amphipathic helix at the N-terminus of VP1. The fact that it was only seen in a few focus classes, when well-ordered Fab was present to support it, argues against it being an artefact of the resolution. Its length is appropriate. As we detail in the supplement (Figure S2), the location of our helix matches the difference density feature that was produced (in Bubeck et al) when the first 30 amino acids were specifically cleaved from symmetric 135S particles, using V8 protease. The susceptibility of poliovirus 135S particles to that sequence-specific cleavage was well established in the 1980s by Fricks et al.

3. In the previous study regarding to poliovirus complexed with soluble Pvr, the cryo-EM reconstruction of the complex can obviously observe the density of Pvr. However, no density was observed in r135 in this study. The authors should explain such difference.

The earlier study reported the structure of the virus receptor complex formed at 4ºC. When the complex is incubated at 37ºC as reported in the present manuscript, the receptor causes the virus to expand which destroys the receptor binding site and causes the receptor to disassociate from the capsid. We understand that this may cause the reader to be confused and thank the reviewer for bringing it our notice. We have therefore added the following line:

Line 264-265: “Note that PVR falls off the virus once expansion has been triggered.”

4. In all the structures of immune complexes, the densities of antibody parts are very weak, therefore the interaction details are not clear. The weakness was not remedied by sub-particle calculation, which should need more check and re-implemented to the best performance. The other reason may raise from the utility of intact antibodies instead of Fab for the preparation of complex. The authors would argue that they could only obtain significant decoration of particles using

the intact monoclonal antibody. But the reason is still unclear. It strongly recommended that Fab generation for sample preparation would improve the resolution of the complex and might visualize some interesting parts, such as the missing VP1 N-terminus. C

The reviewer points out that we were not always clear about the reasons for our choices on the experimental details.

We agree with the reviewer that using a Fab, instead of a mAb, would have been desirable; and indeed, it was the first thing that we tried. We now explain:

Lines 658-659: “Complexes with Fab fragments were also attempted did not produce a detectable levels of decoration).”

We note that mAb often bind to their cognate antigen with higher affinity than their Fab fragments. This is especially true if the intact mAb is able to bind to antigenic sites simultaneously, but can also be attributed to the increased probability of a collision between the antibody and the antigen results in a productive interaction due to the presence of two antigen recognition sites in the mAb versus 1 in an Fab even when bivalent binding is not possible.

We believe that the main reason that the mAb density is weak and smeared out is that it binds to a polypeptide chain that is usually flexible. This is now stated explicitly:

Line 194-196: “The weakness of the Fab density is not unexpected, given that the VP1 N-terminus is flexible, and entirely disordered in most non-native poliovirus structures.”

The reviewer also postulates that further calculations or different calculations could reveal better-ordered density for the Fab. One important point needs to be made:

In order for a well-ordered binding site for this Fab to exist on the outside of poliovirus, it would either be necessary for the flexible N-terminal extension of VP1 (close to the residue 42-55 epitope) to have a high-affinity binding site on the poliovirus outer surface (but we know of no reason why such a binding site should exist), or an accidental stabilizing interaction could occur, which is actually seen in the r135+m25 structure where the density for the Fab is actually quite compelling.

5. It’s strange that the particle heat incubations were performed at 39 °C or 37 °C without specified reason. Also, three classes of particles seem derived from different incubation conditions. The author should describe the details and the consideration reasons.

We agree that our choice of conditions, without explanation, could puzzle a reader. The conditions for the formation of the m135 structure were a compromise, since longer incubations and higher temperatures were needed to get the epitope to reversibly exit from the virus interior, and become accessible to the mAb (even at the current conditions, fully a third of the particles are still undecorated native 160S); but longer incubations and higher temperatures also made the 135S state less stable, triggering RNA loss and conversion to 80S (Figure 1 in the main text).

We now include the following:

Line 131-140: “Initial screening of conditions to trap the N-terminus outside the virus using the mAb at 37˚C yielded very few decorated particles (data not shown). Therefore, we performed additional cryo-EM screenings of the virus-mAb complexes for durations of up to 120 mins at temperatures of 38˚ and 39˚C (data not shown). An optimal number of complexes was observed after a 90-minute incubation of the virus at 39˚C with the mAb.”

Lines 656-658: “(Over numerous trials, these conditions of temperature and duration maximized the proportion of mAb-decorated RNA-containing virus particles.

6. Figure 1 was missed in the merged PDF file, which may lead to some new comments.

We apologise for the inconvenience it may have caused. However, this was glitch in the publisher’s system, and we cannot explain what caused it. Figure 1 was communicated to our case handler as soon as we were informed. Thank you for pointing this out.

Reviewer #1: Minor comments:

1. The radially coloured isosurface rendering of the cryo-EM structures needs the colour scale bars throughout the whole figures. Further, the sigma level and icosahedral symmetric axes of cryo-EM density maps should be indicated in the figures and the corresponding legends.

As also suggested by Reviewer #2, scale bars have been added to Figure 1a, 3a, 4a and 5a, and their captions were changed to mention the bar. Sigma level values have also been added to captions of Figure 2,3,4,5 and 6.

2. Page 7, lines 164-165. The resolution unit was typo as “A”. Please check throughout the manuscript.

This has been corrected throughout.

3. Page 12, lines 263-265. As the author described the structure of r135 and h135 was identical, several related comparison results should be provided in supplementary information.

Our description of the two structures as "identical" was a very poor choice of words. We have fleshed this point out by adding the following lines:

Line 272-279: “The r135 and h135 structures were qualitatively similar. There are no instances where several residues in succession are ordered in one model and disordered or differently ordered in the other. Minor differences between the two models include the occasional shift, by one or two residues along the polypeptide chain, where an order-to-disorder transition occurred, and scattered (rather than systematic) differences in the sets of "buildable" residues in native-like or non-native-like conformations (which are listed in Table S2).”

4. Suppl. Table 1, refinement parameters of r135+m25, especially the Poor rotamers and Disallowed Rama. were high. The model building should be improved.

The footnote for Table 1 has now been updated to state:

Line 1143-1147: * The r135+m25 model, which includes placeholders for the Fab and VP4 densities, was calculated with phenix_refine, using its automatic choice of weighting schemes. Other final atomic models were refined with Refmac5, using stronger stereochemical restraints.”

Reviewer #2:

1. The authors claimed that at least part of the VP4 pentamers indeed remain intact in their five 135S-like particles, which conflicts with many previous experimental observations. Theoretically, it makes sense if viral genome remains intact and inside the particle. However, the relative weak densities for the possible VP4 pentamers in their 135S-like particles are not that convincing to conclude this point. It seems to be solid if authors could provide the mass spectrum results from their purified 135S-like particles to demonstrate the existence of VP4.

Here, based on over 30 years of working with the poliovirus 135S particle we take issue with the reviewers confidence in the reliability of claims that VP4 is totally lacking in 135S particles and their suggestion that further biochemical evidence for the presence of VP4 in the particles is needed to support our identification of this density feature as VP4. The widely accepted claim that 135S particles totally lack VP4 is based primarily on early studies of 135S particles of poliovirus and Coxsackie B3 that are eluted from the surface of cells during infection. We note that because of its small size VP4 stains and radiolabels poorly and it is often difficult to detect in gels optimized for visualizing the larger capsid proteins VP1-3, even when present stoichiometric levels in virions, unless the gels are overloaded. However, we also note that the data shown in these early studies actually often contained evidence for the presence of low levels of VP4 in the 135S particles (see for example figures 7 and 8 in reference Crowell and Philipson 1971 J. Virol. 8:509-515). A later careful study by Curry et al. using the same protocol used to produce the h135 structure in the current manuscript clearly showed that preparations of poliovirus 135S particles contain low levels of VP4 (approximately 25% of that contained in virions), and that this residual VP4 remained upon further purification. These results which were cited (ref 17) in the manuscript, and have been confirmed by subsequent studies in our laboratory. We believe the biochemical evidence for the presence of low levels of VP4 in poliovirus 135S is conclusive. Moreover, as noted in our comments to reviewer 1 and in the manuscript, given that VP4 has been shown to be essential for RNA release and therefore infection, the presence of VP4 in the poliovirus 135S particles is further supported by the demonstration that the 135S particles are infections (Curry et al. ref 17 and Huang et al. ref 18). Finally, we note that low levels of residual VP4 were also described in the publication describing the EV D68 structure (ref 19), and that in the paper describing the CA10 structure where the corresponding density was assigned to RNA the lane for the 135S particle in the SDS gel in supplemental figure 1a was not sufficiently loaded to detect low levels of VP4, and, indeed no band for VP4 was visible in the lane for virions where the load level was much higher and VP4 must be present. We have added text to the revised manuscript (**lines 447-458 (as quoted above in this letter)** to make the evidence that VP4 is present in poliovirus 135S particles clearer)

As stated in the response to review #1, given that we have clearly indicated that the assignment of the density feature to VP4 is somewhat speculative, we believe

that the combination of the biochemical evidence, the position of the density feature, and the shape of the density feature make the suggestion that the feature is a pentamer of VP4 is arguably more plausible than suggestions from other studies that the feature is RNA, and certainly sufficiently plausible to put forward in this manuscript as an alternative model to consider in future studies.

We have also added a sentence to the text, partly to address the reviewer's suggestion to run mass spectrum experiments. For the reasons that we list above, VP4 is unquestionably present in the sample, but mass spec would not address the crucial question of where the VP4 is located.

Lines 610-612: "While these data by themselves do not establish whether VP4 remains on the interior or if it is otherwise bound to the capsid surface, they do underscore the presence of VP4 in highly purified 135S particles."

2. In many cases, the focused reconstruction (local reconstruction as well) have advantages in solving problems, such as structural flexibility, binding occupancy, imperfect symmetry and symmetry mismatch, usually yielding higher resolution with 60-fold particle number in their case. The authors should provide some comments on this point.

The reviewer has noticed that we make extensive use of focused reconstruction methods, without having clearly explained their purpose to some of the readers who may be unfamiliar with the methodology.

We have added a line in the main text and cited previous studies in the literature that used methods similar to the ones that we used.

Line 164-166: "Focused classification methods, recently introduced into cryoEM, often succeed in avoiding the averaging of symmetry-related areas that have significantly different density patterns [37-39]."

3. The radius color bar should be provided in the Figure 1a, 3a, 4a and 5a.

We thank the reviewer for the suggestion. As requested, radius color bars have been added to all four panels. The legends for figures have also been updated with the following line:

"Bar indicates the radial range that each color corresponds to."

4. To make it clear, it would be better to label necessary structural elements, like beta strands in figure 2e and 2f.

The beta strands of VP3 (Figure 2f.) have now been labelled to orient the reader.

5. It’s very rare to bind to the exact symmetrical (two-fold) axes for antibodies/receptors, resulting in the low occupancy due to the steric clashes. Be better to comment this. That might be reason why the densities for Fab fragments are so smear.

The reviewer raises an interesting possibility that had concerned us as well. We have results that are relevant to this concern, but did not mention them in the original manuscript, because the results were negative. We now add a sentence in the main text:

Lines 599-602: “Asymmetric focus maps were calculated for cylindrical volumes near the 2-fold axes, to see if any well-ordered Fab binding sites had been smeared out by symmetry-based density averaging; but no such well-ordered sites were seen (data not shown).”

6. Line 164-165, 4.2 Å, not 4.2 A; 6.5 Å, not 6.5 A; (Fig. 1b) not (Fig. 1b.)

Typos corrected, as suggested.

7. Line 173, 2.8Å resolution, should be “2.8 Å resolution”,

Typos corrected, as suggested.

8. Line 259, 3.2Å resolution, should be “3.2 Å resolution”

Typos corrected, as suggested.

9. Line 660-669, regarding the asymmetrical reconstruction, the authors can refer to the optimized sub-particle reconstruction strategy to have a try (Science, 2018, 360 (6384): 48-58; DOI: 10.1126/science.aaz1439)

We thank the reviewer for bringing this paper to our notice. In the suggested paper, the investigators broke up the ASFV particle up into several sub-particles (based on their symmetry relation) and refined the orientations of each of these sub-particles. They also employed a strategy to correct for the defocus gradient that occurs in a particle of large size to obtain a higher-resolution structure of the virus capsid.

Due to the nature of poliovirus biology, and the small size of our virus, our images included numerous well-ordered, highly symmetric features (which were easily visible at up to 2.8 Å resolution), sitting right next to poorly ordered features that were smeared out. This smearing is due to genuine structural diversity, rather than to problems with optics, defocus, or imperfect symmetry in the virus particle, which the suggested software appears designed to address.

Thus, our approach was not geared toward improving resolution, but for classification based on binding occupancy. The software that we actually used in our poliovirus studies (after trying several alternatives) was mainly directed towards identifying which of the locally smeared densities in the icosahedrally symmetric reconstructions could be classified into chemically reasonable alternative conformations.

10. Line 409-410, As in native virus, the center of the cup (near the 5-fold axis) contacts the VP3 beta tube. It's not easy for most readers to see clearly in figure 6. It's better to color VP3 in red to distinguish with VP1.

Figure 6 has been updated to make the VP3 beta barrel more noticeable.

11. Line 548, "incubation with" should be "Incubation with".

We removed the "dot" from the abbreviation "min.", to make it clear that "incubation" does not begin a new sentence.

Line 582: "… externalized via the quasi-3-fold hole during the initial 3-min incubation with receptor at 37˚C,…'

Additional figure changes were made for clarity:

Added "VP3 GH-loop" label to Figure 3b,c

Additional text changes were made for clarity:

- Small additional changes to fix reference numbering (and the addition of 6 references), and typos, errors in grammar or punctuation, and minor wording changes to improve clarity are scattered throughout the manuscript. These additional small changes are not listed explicitly in the Response Letter.

- In line with PLoS Pathogen's policy, references to data not shown at line numbers 135,138,492 and 602 have been deleted.

Thank you again for your time and attention.
